# Immune response to influenza vaccination in ESRD patients undergoing hemodialysis vs. hemodiafiltration

**DOI:** 10.1371/journal.pone.0227719

**Published:** 2020-02-03

**Authors:** Arkom Nongnuch, Wattanachai Ngampongpan, Sirawat Srichatrapimuk, Artit Wongsa, Sutheera Thongpraphai, Chompunuch Boonarkart, Nutaporn Sanmeema, Malinee Chittaganpitch, Prasert Auewarakul, Boonrat Tassaneetrithep, Andrew Davenport, Angsana Phuphuakrat

**Affiliations:** 1 Department of Medicine, Faculty of Medicine Ramathibodi Hospital, Mahidol University, Bangkok, Thailand; 2 Chakri Naruebodindra Medical Institute, Faculty of Medicine Ramathibodi Hospital, Mahidol University, Samut Prakan, Thailand; 3 Center of Research Excellence in Immunoregulation, Faculty of Medicine Siriraj Hospital, Mahidol University, Bangkok, Thailand; 4 Bhumirajanagarindra Kidney Institute Hospital, Bangkok, Thailand; 5 Department of Microbiology, Faculty of Medicine Siriraj Hospital, Mahidol University, Bangkok, Thailand; 6 National Institute of Health, Ministry of Public Health, Nonthaburi, Thailand; 7 UCL Centre for Nephrology, Royal Free Hospital, UCL Medical School, London, England, United Kingdom; Icahn School of Medicine at Mount Sinai, UNITED STATES

## Abstract

**Background:**

On-line hemodiafiltration (HDF) clears more azotemic toxins compared to high-flux hemodialysis (HD). The response to vaccination is impaired in dialysis patients. We wished to determine whether the immune responses to influenza vaccine in dialysis patients treated by HDF were stronger than those treated by HD.

**Materials and methods:**

We conducted a prospective cohort study in chronic dialysis patients during the 2016 and 2017 influenza seasons. All participants received a single standard dose of trivalent influenza vaccine, and we studied the elicited humoral immune response by hemagglutination inhibition test, and cell-mediated immune response by enumeration of lymphocyte cellular markers and proliferation assays.

**Results:**

We immunized 60 end-stage renal disease (ESRD) patients: 42 (70%) treated with HD and 18 patients (30%) with HDF. The median (interquartile range) age was 65.0 (55.0–74.5) years. All patients developed seroprotection to at least one influenza vaccine strain at one month post-vaccination, and did not differ between groups. By logistic regression, age was the only factor independently associated with seroconversion to all vaccine strains (odds ratio 0.89, 95% confidence interval 0.80–0.98; *p* = 0.022). Seroprotection to all vaccine strains was sustained for longer in patients treated with HDF, and the results remained the same after age adjustment. For cellular immune response, patients who seroconverted to all vaccine strains had higher CD38+ T cell subpopulations pre-vaccination. Patients treated by HDF had higher lymphocyte proliferation to circulating influenza A strains.

**Conclusions:**

Seroconversion to all influenza vaccine strains was associated with age. Patients treated with HDF demonstrated seroprotection was sustained for longer compared to those treated by HD and greater lymphocyte proliferation to circulating influenza A strains. These encouraging results for HDF require confirmation in a larger dialysis population.

**Trial registration:**

ClinicalTrial.gov, NCT04122222.

## Introduction

The retention of waste products of metabolism in patients with chronic kidney disease (CKD) leads to impaired function of the immune system; both innate and adaptive immunity [1–3]. There have been reports directly linking the accumulation of azotemic toxins, non-protein nitrogenous compounds in blood such as blood urea nitrogen, creatinine, phenols, advanced glycosylation end products, and an impaired or dysregulated immune response [4–6], including reduced neutrophil phagocytosis [7], as well as reduced numbers of circulating monocytes [8], T-lymphocytes [9], and B-lymphocytes [10]. This immune dysregulation increases the risk of infection, and as deaths from cardiovascular disease continue to fall, infection is an increasing cause of death in CKD patients. Compared to the general population, CKD patients not only have an approximate two-fold increase in the incidence of pulmonary infections [11], but also have infections of greater severity as evidenced by higher hospitalization rates, complications during admission and mortality [12, 13]. Previous studies have reported a 14–16 fold increase in mortality from respiratory tract infections (RTIs) in hemodialysis patients compared to the general population [14].

Although hemodialysis is an established treatment for end-stage renal disease (ESRD), it is a life-sustaining treatment; with a long-term survival worse than those of several of the more common solid organ malignancies [15]. The high mortality may well be multifactorial; reflecting underlying co-morbidity, chronic volume overload, retention of azotemic toxins, chronic inflammation, and malnutrition. High-flux hemodialysis (HD) increases the range of middle-sized azotemic toxin clearance compared to the original low-flux dialyzers, but the previous study did not demonstrate any reduction in mortality from infection in patients treated by HD [16]. More recently, on-line hemodiafiltration (HDF) has been introduced into clinical practice, and the addition of convection can potentially lead to a substantially greater removal of middle-sized azotemic toxins compared to HD. Previous studies have reported that HDF provided greater clearance of inflammatory cytokines compared to HD, and resulted in lower C-reactive protein (CRP) concentrations [17], although overall infection rates were comparable [18].

There have been several studies investigating the response to hepatitis B vaccination, and the rate of seroconversion is lower in patients with more advanced CKD compared to those with earlier stages, suggesting that retention of azotemic toxins impairs the immune response to vaccination [19]. This is supported by a study demonstrating a greater seroconversion rate in dialysis patients treated with high flux compared to low flux hemodialysis [20]. We therefore wished to determine whether HDF, by increasing the spectrum of middle-sized azotemic toxin clearance, improved the immune response to influenza vaccination compared to HD.

## Materials and methods

### Study design and participants

A prospective cross-sectional cohort study was conducted in chronic dialysis patients at Ramathibodi Hospital and Bhumirajanagarindra Kidney Institute Hospital during the 2016 and 2017 influenza seasons. We recruited ESRD patients aged 18 years or older, who had been treated for more than one month of either thrice weekly HDF or conventional HD, with a session dialyzer urea clearance (Kt/Vurea) of 1.2 or greater. Since convection volume in HDF determines the efficacy of azotemic toxin removal and minimum adequate convection volume is between 17–21 L/session [21], we included patients who had adequate convection volume of approximately 20 L/session. We excluded patients who had received any vaccination within the previous four weeks, or influenza vaccination within six months. Additionally, we excluded any patient who reported upper respiratory tract symptoms within three days prior to the study vaccination and those with a history of allergy to influenza vaccine or egg, thrombocytopenia, in receipt of immunosuppressant medications, chemotherapy, or had immunodeficiency.

### Ethics statement

The study protocol was reviewed and approved by the Ethical Clearance Committee on Human Right Related to Research Involving Human Subjects of the Faculty of Medicine Ramathibodi Hospital, Mahidol University (ID 07-59-14), and in accordance with the principles of the Declaration of Helsinki. Written informed consent was obtained from all participants.

### Influenza immunization

All participants received a single standard dose of inactivated trivalent influenza vaccine: Influvac^®^ vaccines (Abbott, Weesp, The Netherlands), intramuscularly. The 2016 vaccine contained 15 mcg of hemagglutinin per strain of A/California/7/2009, X-181 (H1N1), A/Hong Kong/4801/2014, X-263B (H3N2), and B/Brisbane/60/2008, wild type. The 2017 vaccine contained 15 mcg of hemagglutinin per strain of A/Singapore/GP1908/2015, IVR-180 (H1N1), A/Hong Kong/4801/2014, NYMC X-263B (H3N2) and B/Brisbane/60/2008, wild type.

### Demographics and clinical data

Relevant information was extracted from hospital medical records; including demographics, dialysis vintage, dialysis adequacy (Kt/Vurea), comorbid diseases, current drug treatment. Blood was sampled predialysis at the time of inclusion and analyzed for complete blood count, urea nitrogen, creatinine, albumin, calcium, phosphorus, parathyroid hormone, and serum ferritin.

The participants were asked about clinical respiratory tract infection symptoms thrice weekly when attending for dialysis treatments throughout the study period. Influenza infection was confirmed by rapid test or PCR-based respiratory viral identification (xTAG^®^ respiratory viral panel, Luminex, Austin, TX, US).

### Blood sampling

Blood samples were taken to assess the immune response to vaccination pre-vaccination (month 0) and post-vaccination at months 1, 6, and 12. Serum was separated, and stored at -20°C until analysis. For peripheral blood mononuclear cell (PBMC) isolation, 20 mL of peripheral blood was collected into sodium heparin tubes. PBMCs were then isolated by gradient centrifugation using Histopaque^®^ (Sigma-Aldrich, Saint Louis, MO, US) according to the manufacturer’s instructions.

### Influenza antibody titers

Antibody titers were determined by a hemagglutination inhibition (HI) assay. Serum samples were tested against A/California/7/2009 A(H1N1)pdm09-like, A/Hong Kong/4801/2014-like (H3N2), B/Brisbane/60/2008-like for the 2016 season and A/Michigan/45/2015-like (H1N1)pdm09, A/Hong Kong/4801/2014-like (H3N2), B/Brisbane/60/2008-like for the 2017 season. Briefly, serum samples were treated with receptor-destroying enzyme (RDE; Denka Seiken, Tokyo, Japan): 300 μL of RDE was added to 25 μL of serum and incubated overnight at 37°C, followed by adsorption with test red blood cells and heat inactivation to eliminate nonspecific inhibitors and nonspecific agglutinators. The treated serum was then serially diluted, and 8 hemagglutination (HA) units / 50 μl of the virus were added. The mixture was then incubated at 25°C for 30 minutes. Inhibition of hemagglutination determined after incubating with 0.5% goose erythrocytes (provided by National Laboratory Animal Center, Mahidol University) at 25°C for 30 minutes. HI titers were recorded as the inverse of the highest antibody dilution that inhibited hemagglutination.

Seroconversion was defined as either a pre-vaccination HI antibody titer ≤1:10 and a post-vaccination titer ≥1:40 or a pre-vaccination titer >1:10 and a four-fold or greater increase in the post-vaccination titer. The seroprotection rate was defined as a HI antibody titer of 1:40 or more.

### Immunophenotyping

The frozen PBMCs stored in liquid nitrogen were thawed and washed in RPMI 1640 medium (Gibco, Carlsbad, CA, US) supplemented with 10% fetal bovine serum (Merck Millipore, Burlington, MA, US) and containing DNase I (Sigma-Aldrich, Saint Louis, MO, US). Dead cells were excluded by staining with LIVE/DEAD^™^ Fixable Aqua Stain (Invitrogen, Carlsbad, CA, US) at 4°C for 15 minutes. Then, PBMCs were washed and stained with 50 μL monoclonal antibody cocktail against surface markers for T lymphocyte population including, Pacific Blue^™^ anti-human CD3 antibody (1:80), Brilliant Violet 605^™^ anti-human CD45RO antibody (1:80), Brilliant Violet 650^™^ anti-human CD127 (IL-7Rα) antibody (1:80), Brilliant Violet 711^™^ anti-human CD28 antibody (1:80), Brilliant Violet 785^™^ anti-human CD197 (CCR7) antibody (1:160), PE/Dazzle^™^ 594 anti-human CD45RA antibody (1:160), PE/Cy5 anti-human CD25 antibody (1:160), PE/Cy7 anti-human CD38 antibody (1:160), APC anti-human CD57 antibody (1:80), Alexa Fluor® 700 anti-human CD8 antibody (1:80), APC/Cy7 anti-human CD4 antibody (1:160) (BioLegend, San Diego, CA, US), and BV510 mouse anti-human CD235ab antibody (1:160), PE mouse anti-human CD279 (PD-1) antibody (1:40) (BD Biosciences, San Jose, CA, USA) followed by intracellularly stained transcription factor Alexa Fluor® 488 anti-mouse/rat/human FOXP3 antibody (1:80) (BioLegend, San Diego, CA, USA). Antibodies used for B lymphocyte staining include: Brilliant Violet 711^™^ anti-human CD27 antibody (1:80), Brilliant Violet 785^™^ anti-human HLA-DR antibody (1:160), PE-CF594 mouse anti-human CD19 antibody (1:160), PE/Cy5 anti-human CD3 antibody (1:160), PE/Cy7 anti-human CD38 antibody (1:160), APC anti-human CD24 antibody (1:80), PE mouse anti-human CD279 (PD-1) antibody (1:40) (BD Biosciences, San Jose, CA, USA). Stained cells were analyzed via BD LSRFortessa (BD Biosciences, San Jose, CA, US). Data were analyzed using FlowJo software (Tree Star, Ashland, OR, US).

### Influenza viruses and inactivation

Circulating influenza viruses in Thailand between 2016–2018 were used to study the cell proliferation assay. Influenza A viruses [A/Nonthaburi/140/2016 (A/California/7/2009(H1N1)pdm09-like virus), A/Ayutthaya/24/2017 (A/Hong Kong/4801/2014(H3N2)-like virus), A/Chanthaburi/291/2017 (A/Michigan/45/2015(H1N1)pdm09-like virus), and A/Tak/240/2017 (A/Singapore/INFIMH-16-0019/2016(H3N2)-like virus)] and Influenza B viruses [B/Chiang Mai/21/2017 (B/Brisbane/60/2008-like virus), and B/Nan/451/2017 (B/Phuket/3073/2013-like virus; B/Yamagata/16/88 lineage)] were grown in Madin Darby canine kidney (MDCK) cells, which were kindly provided by Professor Malik Peiris, University of Hong Kong. The cells were cultured in Minimum Essential Medium (MEM, Gibco, Carlsbad, CA, US) containing 1 μg/mL of L-1-tosylamide-2-phenylethyl chloromethyl ketone (TPCK)-treated trypsin (Sigma-Aldrich, Saint Louis, MO, US). The infections were performed at 37°C. The cell culture supernatants containing viruses were collected 48 hours after infection, and were centrifuged for 15 minutes at 300 g, 4°C. The supernatants were stored at -70°C. Virus titers were quantified by plaque assay. The cells were seeded in 12-well plates and incubated overnight at 37°C. Cells were then washed with MEM (Gibco, Carlsbad, CA,US) containing 1 ug/mL of L-1-tosylamide-2-phenylethyl chloromethyl ketone (TPCK)-treated trypsin (Sigma-Aldrich, Saint Louis, MO, US). One hundred microliters of a 10-fold dilution of the virus were added and the cells were incubated in a 5% CO_2_ incubator at 37°C with shaking every 15 minutes for 1 hour. The inoculums were then removed and cells were overlaid with MEM containing 1% low-melting point agarose gel (Promega, Madison, WI, US). After being incubated for 48 hours, the cells were fixed with 10% formaldehyde for 1 hour, and then stained with 1% crystal violet for 20 minutes. Virus stocks were ultraviolet (UV)-inactivated for 30 minutes prior to co-culture with PBMC.

### Lymphocyte proliferation assay

After thawing, PBMCs were stained with carboxyfluorescein succinimidyl ester (CSFE, Invitrogen, Carlsbad, CA, US) in the final concentration of 1 μM. The stained cells were co-cultured with UV-inactivated influenza virus obtained from the culture supernatants described in the previous section or mitogens phytohemagglutinin (positive control, Invitrogen, Carlsbad, CA, US) for 120 hours at 37°C in a humidified 5% CO_2_ atmosphere. Stimulated cells were stained with Pacific Blue^™^ anti-human CD3 antibody (1:80), APC/Cy7 anti-human CD4 antibody (1:160), Alexa Fluor® 700 anti-human CD8 antibody (1:80), and PE-CF594 mouse anti-human CD19 antibody (1:160) (BioLegend, San Diego, CA, US) for 15 minutes at room temperature, and examined via BD LSRFortessa (BD Biosciences, San Jose, CA, US). Negative control was unstimulated lymphocytes of the same patients at prevaccination. Individual negative control was used to normalize the percentage of lymphocyte proliferation.

### Statistical analysis

Data were analyzed for normality and descriptive statistics presented as a number (percent) for categorical variables and median (interquartile range; IQR) for continuous variables. Chi-square or Fisher’s exact test was used for categorical variables. Mann-Whitney *U*-tests were used to compare continuous variables. Logistic regression was used to determine the factors associated with seroconversion to all three vaccine strain viruses. Variables that presented a *p*-value <0.2 from univariate logistic regression were considered in a multivariate logistic regression model. Odds ratio (OR) and its 95% confidence interval (CI) were estimated. Outcomes with repeated measurement were compared using multilevel mixed-effects linear regression model. All statistical analyses were performed using the Stata statistical software version 15.1 (StataCorp, College Station, TX, US).

## Results

### Patient characteristics

We studied 60 dialysis patients; 42 (70.0%) treated by HD and 18 (30.0%) by HDF, respectively. Thirty-one (51.7%) were female, with a median age (interquartile range; IQR) of 65.0 (55.0–74.5) years. Median body mass index (BMI) was 22.8 (20.1–26.5) kg/m^2^, and median duration of dialysis treatment was 4.3 (3.2–8.4) years. The most common causes of ESRD were diabetes and hypertension. Demographic data of the patients are reported in Table 1. Patients treated by HDF were younger than those treated by HD. There were no statistically significant differences between the two groups in sex, BMI, underlying diseases, and dialysis adequacy. Blood urea nitrogen (BUN) and serum creatinine (Cr) were significantly lower in HD patients. There were no statistically significant differences between the two groups regarding complete blood count, and other blood chemistry tests.

**Table 1:** Patients’ characteristics.

	Hemodialysis (n = 42)	Hemodiafiltration (n = 18)	*p*-value
**Demographics**			
Age, years (IQR)	67.5 (57.0–78.0)	56.0 (50.0–70.0)	0.025
Female	22 (52.4%)	9 (50.0%)	>0.999
Body mass index, kg/m^2^ (IQR)	22.7 (20.1–26.4)	24.6 (20.0–27.0)	0.580
Dialysis vintages, years (IQR)	3.9 (3.1–6.6)	5.3 (3.5–10.5)	0.095
**ESRD causes**			
Diabetic nephropathy	24 (57.1%)	6 (33.3%)	0.158
Hypertensive nephropathy	13 (31.0%)	10 (55.6%)	0.089
Glomerulonephritis	2 (4.8%)	1 (5.6%)	>0.999
Polycystic kidney disease	2 (4.8%)	1 (5.6%)	>0.999
Others	3 (7.1%)	2 (11.1%)	0.631
**Underlying diseases**			
Diabetes mellitus	27 (64.3%)	7 (38.9%)	0.091
Hypertension	40 (95.2%)	18 (100.0%)	>0.999
Dyslipidemia	24 (57.1%)	11 (61.1%)	>0.999
CAD	12 (28.6%)	4 (22.2%)	0.755
Congestive heart failure	4 (9.5%)	2 (11.1%)	>0.999
Atrial fibrillation	3 (7.1%)	0 (0.0%)	0.547
CVA	5 (11.9%)	0 (0.0%)	0.309
COPD	2 (4.8%)	0 (0.0%)	>0.999
Cirrhosis	1 (2.4%)	0 (0.0%)	>0.999
Chronic hepatitis B	2 (4.8%)	1 (5.6%)	>0.999
Malignancy	3 (7.1%)	1 (5.6%)	>0.999
**Intra-HD hypotension on day 0**	5 (12.2%)	3 (16.7%)	0.690
**Kt/Vurea**	1.9 (1.6–2.2)	1.7 (1.6–2.1)	0.577
**Medication**			
Iron	16 (38.1%)	10 (55.6%)	0.261
Erythropoietin dose, IU/week	8,000 (6,000–12,000)	6,000 (5,000–10,000)	0.266
Statin	23 (56.1%)	12 (66.7%)	0.568
**Laboratory parameters**			
Hemoglobin, g/dL	10.6 (9.9–11.5)	11.0 (10.4–11.7)	0.269
White blood cells, /mm^3^	6.4 (5.2–8.1)	6.5 (5.4–7.0)	0.872
Platelets, /mm^3^	184.5 (153.0–232.0)	179.5 (147.0–212.0)	0.910
Blood urea nitrogen, mg/dL	55.5 (45.0–68.0)	77.0 (62.0–84.0)	0.009
Creatinine, mg/dL	9.2 (7.5–10.8)	11.0 (8.6–13.8)	0.028
Hemoglobin A1c, %	5.6 (5.1–7.0)	6.5 (5.5–7.7)	0.345
Sodium, mmol/L	138.0 (136.0–140.0)	139.0 (137.0–139.0)	0.284
Potassium, mmol/L	4.4 (4.1–4.6)	4.6 (4.1–5.2)	0.238
Chloride, mmol/L	97.0 (95.0–99.0)	97.0 (96.0–99.0)	0.394
Bicarbonate, mmol/L	22.0 (20.0–23.0)	21.5 (20.0–24.0)	0.639
Calcium, mg/dL	8.7 (8.4–9.0)	9.0 (8.4–9.2)	0.405
Phosphate, mg/dL	4.1 (2.9–5.4)	4.9 (3.4–5.2)	0.345
Albumin, g/dL	3.6 (3.3–3.9)	3.4 (3.2–3.5)	0.106
LDL-C, mg/dL	88.5 (60.0–105.0)	95.0 (54.0–104.0)	0.955
Triglyceride, mg/dL	105.0 (78.0–155.0)	104.5 (79.0–127.0)	0.968
iPTH, pg/mL	416.0 (197.0–566.0)	318.5 (144.0–614.0)	0.542
Ferritin, ng/mL	458.0 (253.0–708.0)	365.5 (260.0–540.0)	0.223
Transferrin saturation, %	27.0 (21.0–34.0)	24.7 (23.0–32.0)	0.458
Beta 2 microglobulin, mcg/mL	23.0 (19.1–28.2)	22.3 (19.2–24.4)	0.329
C-reactive protein, mg/dL	0.31 (0.10–0.75)	0.18 (0.10–0.36)	0.482

**Abbreviations:** CAD, coronary artery disease; COPD, chronic obstructive pulmonary disease; CVA, cerebrovascular accident; ESRD, end-stage renal disease; HD, hemodialysis; iPTH, intact parathyroid hormone; IQR, interquartile range; LDL-C, low-density lipoprotein cholesterol level; RRT, renal replacement therapy

Local adverse effects were reported as pain at the site of infection in eight (13.3%) and one (1.7%) swelling at the injection site. The most commonly reported systemic symptoms were myalgia and dizziness each in 5 patients (8.3%), 3 (5.1%) cases of ever requiring antipyretic drugs, and one case of nausea (1.7%).

During the study, there was one documented case of influenza, which occurred in a 50-year-old man in the HD group, one month after vaccination. Multiplex RT-PCR revealed this to be influenza A H1N1 2009. He had not seroconverted and had no seroprotection to H1N1 2009 when tested at one month post-vaccination. He was treated with oseltamivir for five days and recovered without any influenza-related complications.

**Fig 1:**
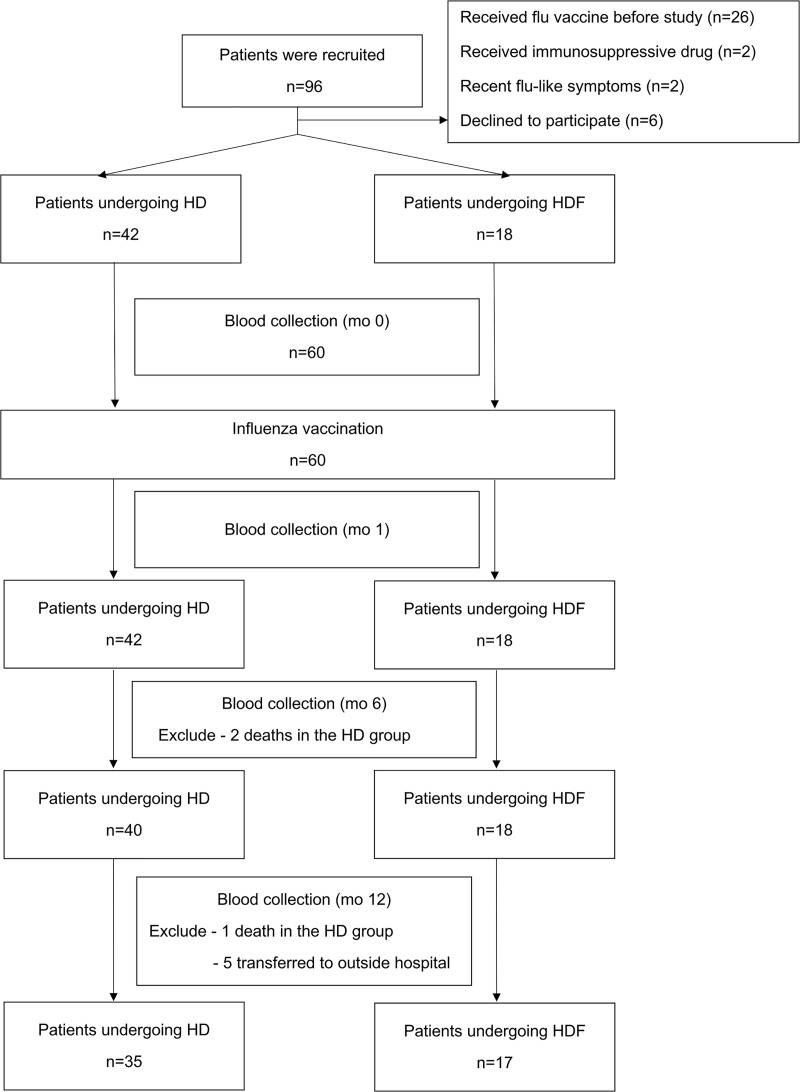
Study enrollment and follow-up through month 12.

The study flow diagram is shown in Fig 1. During the study, there were three deaths (5.0%), all unrelated to vaccination and occurred in the HD group. The first patient, a 78-year-old man with diabetes mellitus (DM), hypertension, and a previous stroke, died at home with unknown cause three months after study entry. A 53-year-old woman with DM died after seven months after study inclusion, following an acute stroke. The third patient, a 78-year-old man with hypertension and dyslipidemia died from candida septicemia one month after study entry.

### Humoral immune response to influenza vaccine

**Fig 2:**
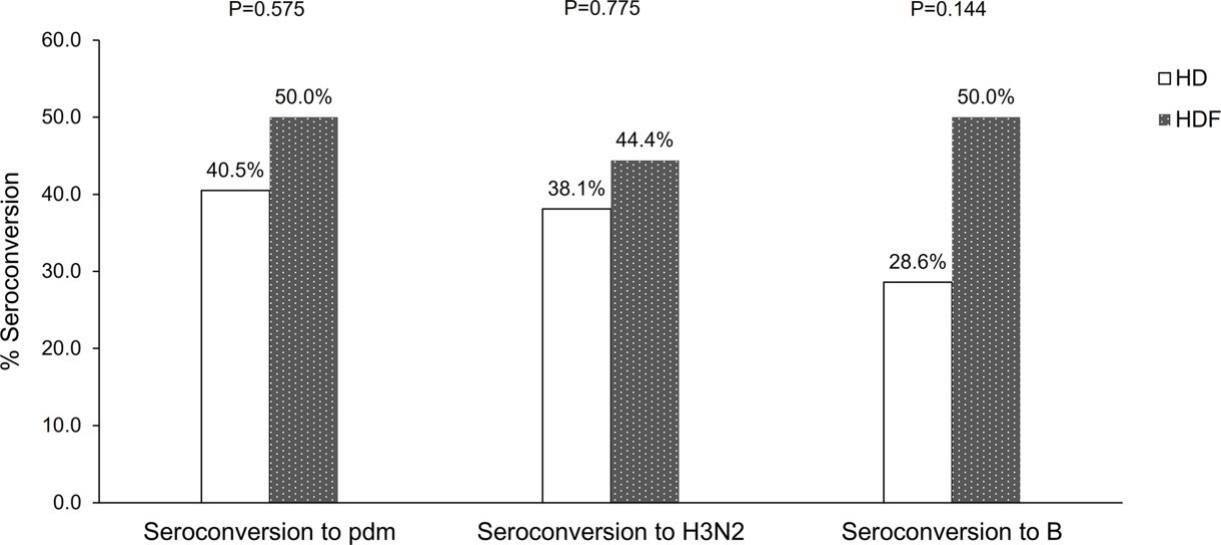
Proportion of participants who had seroconversion at month 1 to influenza H1N1 pandemic, H3N2, and B.

**Fig 3:**
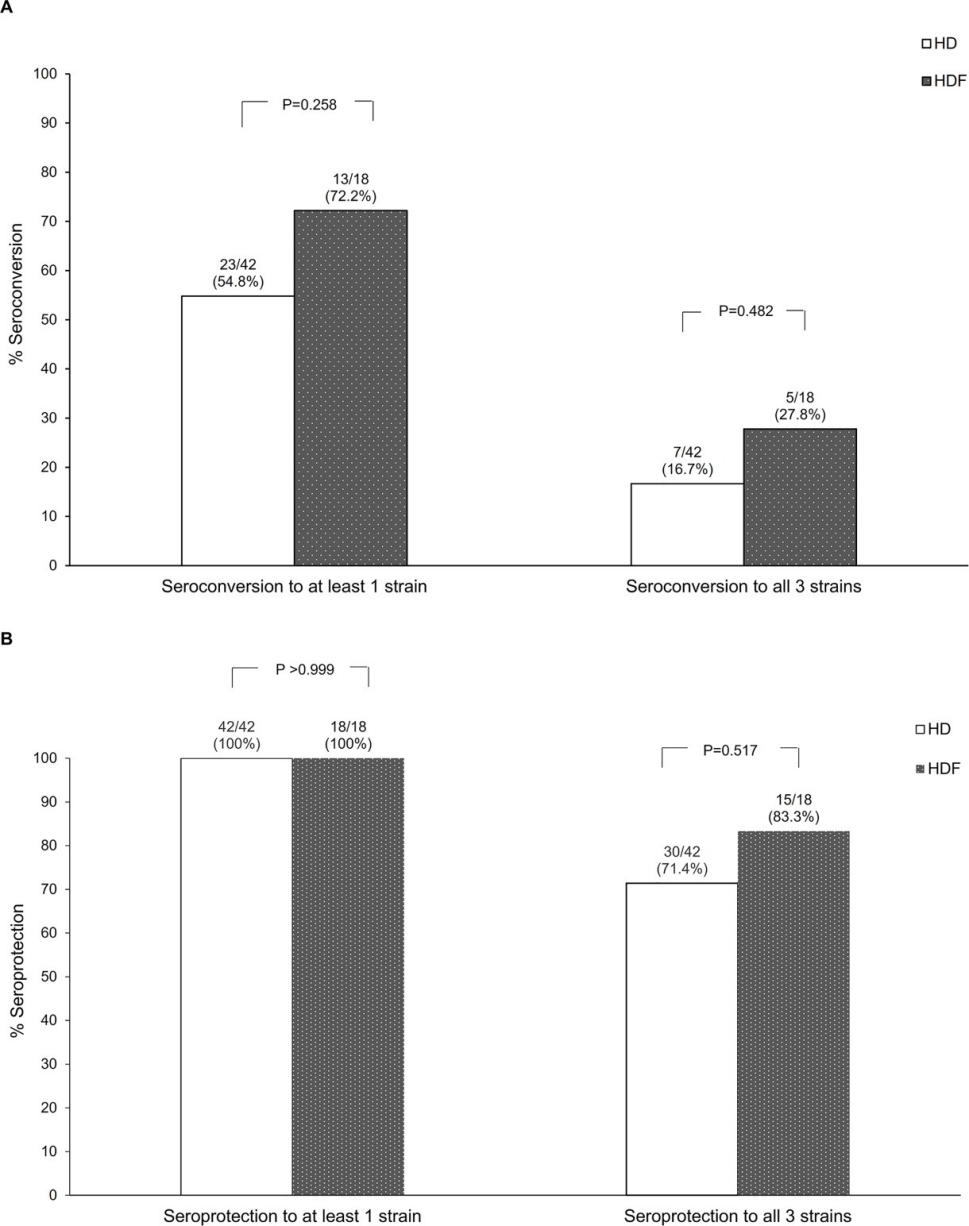
Proportion of participants who seroconverted to at least one or all of the three vaccine strains at 1 month post-vaccination (A), and proportion of participants with seroprotection to at least 1 or all three of the vaccine strains at 1 month post-vaccination (B).

**Fig 4:**
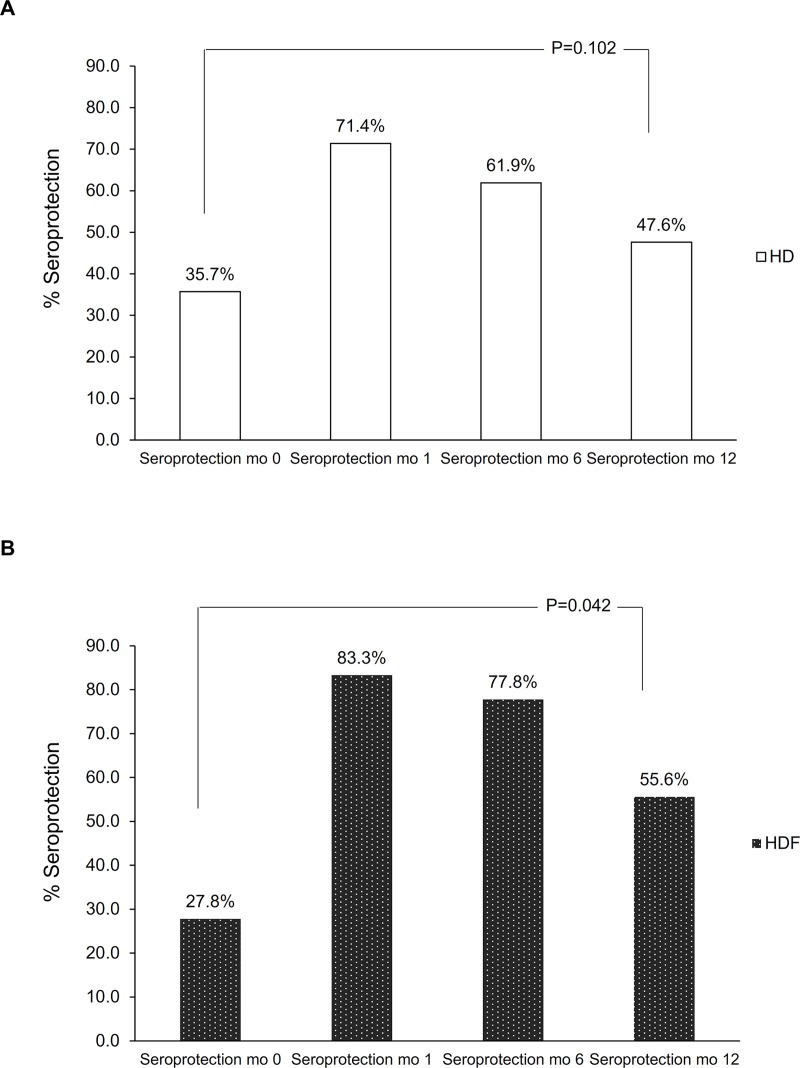
Proportion of participants who had seroprotection to all vaccine strains pre-vaccination (mo 0), and post-vaccination at months 1 (mo 1), 6 (mo 6), and 12 (mo 12) (A) HD, (B) HDF.

Seroconversion to influenza vaccine strain H1N1pdm, H3N2, and B at month 1 post-vaccination was not significantly different between patients treated by HD and those treated by HDF (Fig 2; *p =* 0.575, 0.775, and 0.144, respectively). At month 1 post-vaccination, all patients had developed seroprotection to at least one influenza strain (S1 Fig). There was no significant difference in both seroconversion and seroprotection, either to at least one strain or to all three strains, between the two groups (Fig 3). Compared to month 0, the proportion of patients with seroprotection was significantly higher after influenza vaccination at months 1 and 6 in both groups [odds ratio (OR) HD 64.3, 95% confidence interval (CI) 12.5 to 331.0; *p* <0.001, and OR HDF 24.6, 95% CI 5.9 to 102.8; *p* <0.001). Compared to month 0, the proportion of patients with seroprotection at month 12 remained significant for the HDF group (OR 12.2, 95% CI 1.1 to 136.0; *p* = 0.042), but not for the HD group (OR 3.2, 95% CI 0.8 to 12.8; *p* = 0.102) (Fig 4).

We investigated potential factors associated with seroconversion to all three strains (Table 2). On univariate logistic regression, factors with a *p* <0.2 included age, gender, BMI, dialysis vintage, low-density lipoprotein (LDL) cholesterol level, and ferritin. Age remained the only factor associated with seroconversion on multivariate logistic to all three influenza strains [odds ratio (OR) 0.89, 95% confidence interval (CI) 0.80–0.98; *p* = 0.022]. We also analyzed proportions of patients with seroprotection at month 12 compared to month 0 after age adjustment. This proportion remained significant for the HDF group, but not the HD group (OR 11.6, 95% CI 1.1–124.7; *p* = 0.044, and OR 3.2, 95% CI 0.8–12.8; *p* = 0.102, respectively).

**Table 2:** Univariate and multivariate analysis of factors associated with seroconversion to all three vaccine strains.

Factors	Univariate		Multivariate	
	OR (95% CI)	*p*-value	OR (95% CI)	*p*-value
Age, per 1 year increase	0.94 (0.89–0.99)	0.029	0.89 (0.80–0.98)	0.022
Female	0.23 (0.06–0.99)	0.049	0.24 (0.02–2.97)	0.264
Body mass index	0.90 (0.77–1.05)	0.171	0.74 (0.53–1.04)	0.083
Dialysis vintage	1.12 (0.99–1.27)	0.062	1.14 (0.93–1.41)	0.213
LDL-C	0.98 (0.96–1.00)	0.094	0.95 (0.91–1.00)	0.050
Ferritin	1.00 (0.99–1.00)	0.100	1.00 (0.99–1.00)	0.263

**Abbreviations:** CI, confidence interval; LDL-C, low-density lipoprotein cholesterol level; OR, odds ratio

### Immunophenotyping of lymphocytes

**Fig 5:**
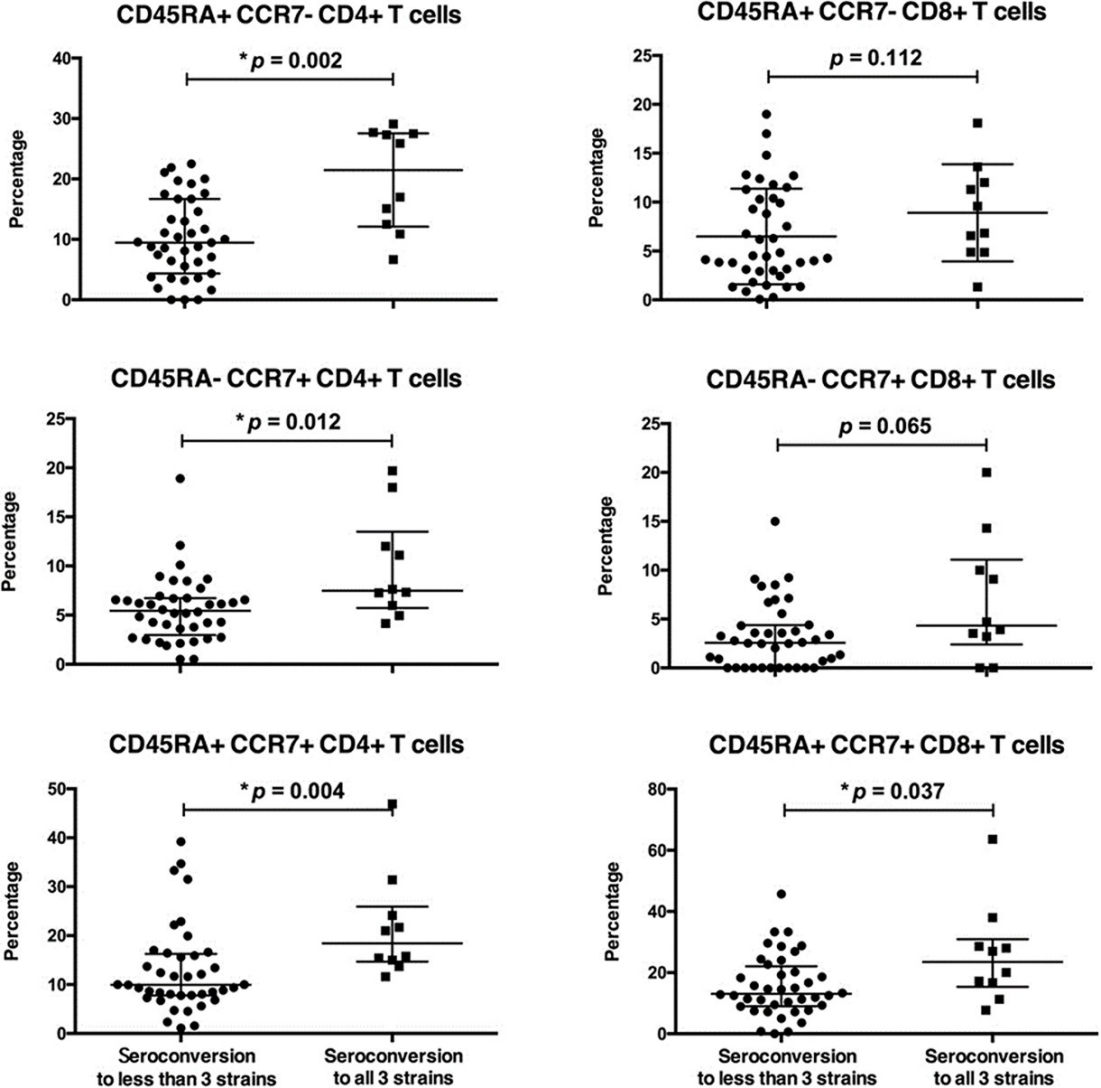
CD38+ T cell subpopulations at pre-vaccination. Effector memory (TEM, CD45RA+CCR7-), central memory (TCM, CD45RA-CCR7+), and naive (CD45RA+CCR7+) T cell subpopulations are shown.

We compared those patients who had seroconversion to all three vaccine strains to those who had seroconversion to two or fewer strains, patients who demonstrated seroconversion to all three vaccine strains had significantly higher CD38+CD4+ T cell and CD38+CD8+ T cell subpopulations pre-vaccination (Fig 5).

**Fig 6:**
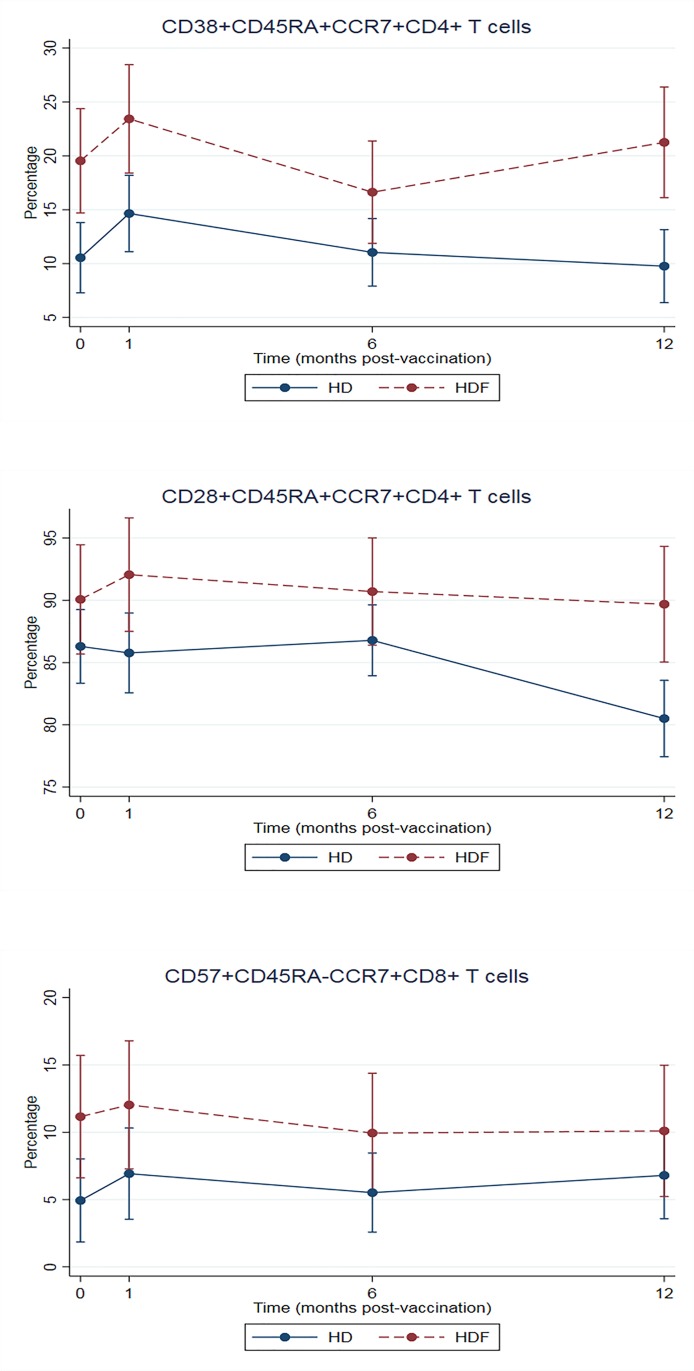
Age-adjusted percentage of T cell subpopulations at pre-vaccination and post-vaccination.

Enumeration of CD4+, CD8+ and regulatory T cell (Treg; CD3+CD4+CD25+FoxP3+) showed no difference either pre-vaccination or post-vaccination between HDF and HD patients. Further analysis of subpopulations of CD4+ and CD8+ T cells showed that age-adjusted percentage of CD38+CD45RA+CCR7+CD4+ T cells, CD28+CD45RA+CCR7+CD4+ T cells, and CD57+CD45RA-CCR7+CD8+ T cells were higher in those patients treated by HDF (Fig 6 and Table 3). There were no significant differences in the percentages of other T cell subpopulations between the HD and the HDF groups.

Enumeration of B cell subpopulations, including memory B cells (S2 Fig), showed no significant difference between HDF and HD patients.

**Table 3:** Mean change in subpopulations of T cells from baseline.

	Change^a^ (95% CI)			Change^a^ (95% CI)			
	HD at month 1	HD at month 6	HD at month 12	HDF at month 0	HDF at month 1	HDF at month 6	HDF at month 12
CD38+CD45RA+CCR7+CD4+ T cells	4.11* (0.66–7.55)	0.50 (-2.56–3.56)	-0.78 (-4.07–2.51)	8.12* (2.06–14.17)	12.01* (5.80–18.22)	5.21 (-0.75–11.16)	9.83* (3.57–16.09)
CD28+CD45RA+CCR7+CD4+ T cells	-0.52 (-3.59–2.54)	0.49 (-2.24–3.21)	-5.79* (-8.72–2.87)	3.55 (-1.94–9.04)	5.53 (-0.09–11.16)	4.18 (-1.23–9.58)	3.16 (-2.51–8.83)
CD57+CD45RA-CCR7+CD8+ T cells	1.99 (-1.57–5.55)	0.59 (-2.58–3.75)	1.87 (-1.53–5.26)	6.93* (1.25–12.62)	7.81* (1.95–13.66)	5.71* (0.13–11.29)	5.87 (-0.04–11.79)

**Abbreviations:** CI, confidence interval; HD, hemodialysis; HDF, hemodiafiltration

^a^Multilevel mixed-effects linear regression, reference is T cell subpopulations at baseline of HD arm

*p <0.050

### Cellular immune response

**Fig 7:**
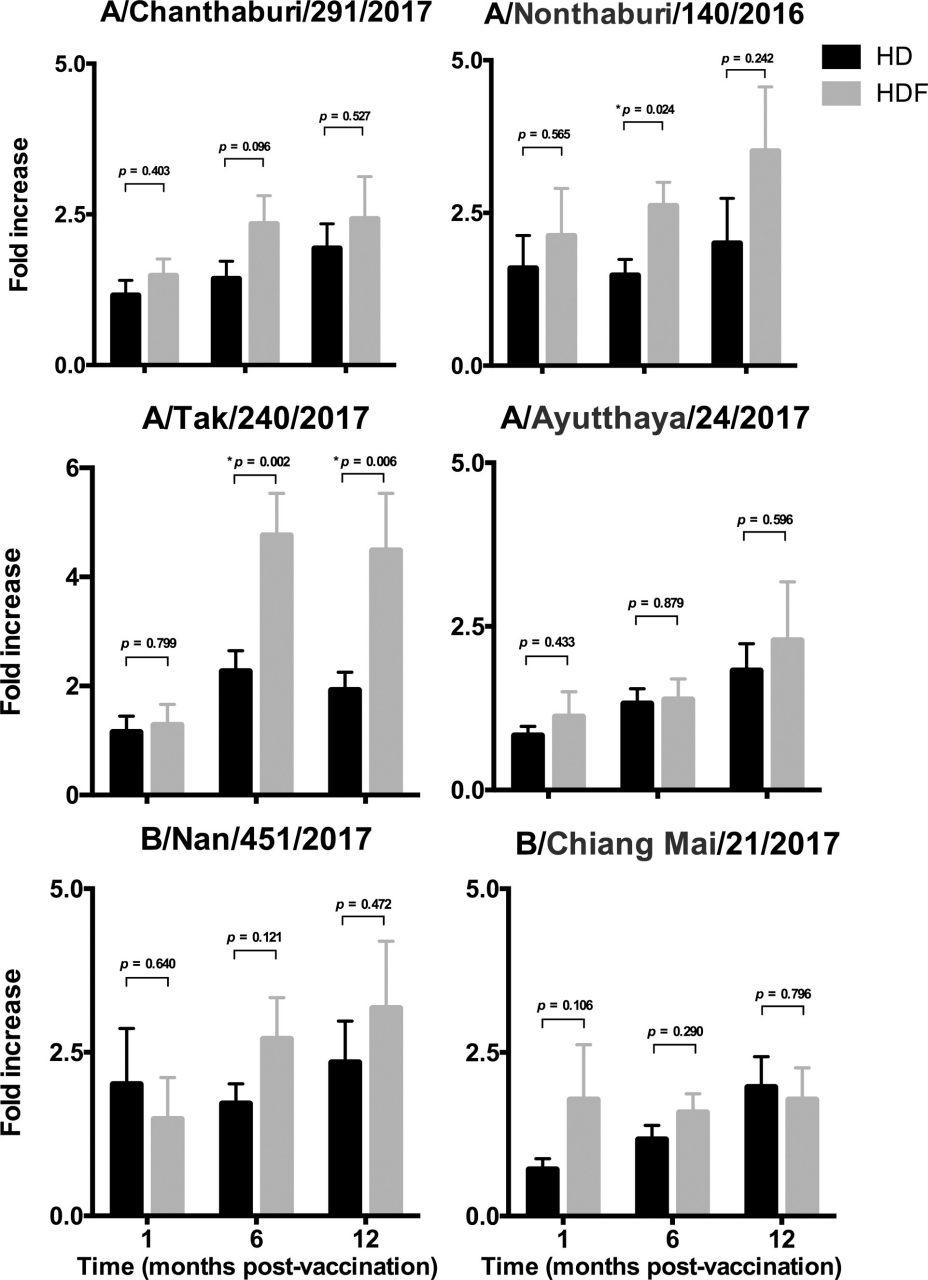
Lymphocyte proliferation assays to circulating strains of influenza viruses. Fold increases of lymphocyte proliferation as compared to pre-vaccination are shown.

Recall specific memory lymphocyte response to circulating strains of influenza viruses was determined by lymphocyte proliferation assays. Higher lymphocyte proliferation to A/Tak/240/2017 strain (A/Singapore/INFIMH-16-0019/2016(H3N2)-like virus) was demonstrated in patients treated by HDF compared to those treated by HD at months 6 and 12- post-vaccination (*p* = 0.002 and *p* = 0.006 respectively). Higher lymphocyte proliferation to A/Nonthaburi/140/2016 (A/California/7/2009(H1N1)pdm09-like virus) was also demonstrated at month 6 post-vaccination (*p* = 0.024) in the HDF patients. There was no significant increase in lymphocyte proliferation to other influenza strains between the HDF and HD groups (Fig 7 and S3 Fig).

## Discussion

A previous study reported that treatment with HDF resulted in greater clearance of inflammatory cytokines compared to HD [17]. The accumulation of azotemic toxins has a major role in determining the morbidity and mortality in ESRD dialysis patients. As HDF increases middle molecule azotemic toxin clearances compared to HD, HDF may potentially reduce systemic inflammation, and one study has reported a reduction in CRP levels with HDF compared to HD [21]. A reduction in systemic inflammation could potentially lead to an improved immunologic response [17, 22, 23]. However, the immunological response elicited by vaccines in patients treated by HDF has not been well established. We therefore wished to study if there were differences in humoral and cell-mediated immune responses to influenza vaccine in dialysis patients treated by HD and HDF.

In terms of the humoral immune response, we demonstrated that there were no significant differences in seroconversion at one month post-vaccination by HI assay. Our seroconversion rates to influenza vaccination were comparable to those previously reported in HD patients [24]. However, we observed the patients treated by HDF had a longer duration of seroprotection compared to those treated by HD at 12 months. An earlier report showed that post-vaccination HI titers waned in older adults, and were not sustained after 12 months [25]. It has been suggested that ESRD dialysis patients have premature aging of their immune system, as young dialysis patients have phenotypic and functional changes in their immune cells similar to those found in healthy elderly individuals [26].

In multivariate analysis, younger age was associated with seroconversion to all three vaccine strains. This supports previous studies which have also reported that age was a determinant factor in the immune response to influenza vaccine [24, 27]. In our study, the HDF cohort was younger, with higher pre-dialysis BUN and creatinine, although marginally longer dialysis vintage, potentially suggesting that this group might have been healthier, although co-morbidity was similar to the HD patients. Although HDF would be expected to reduce β2 microglobulin concentrations compared to HD, there was no difference between groups. This probably reflects that the HDF cohort had been treated by dialysis for longer and had less residual renal function. As this study was the observational study, selection bias was inevitable. Technically, HDF with high convection volume tended to be assigned to the patients who have good vascular access flow since it needs very good blood flow in order to achieve high convection volume. Thus, according to physician discretion, the patients who were younger and had low atherosclerosis risks tended to undergo HDF.

We also assessed cell-mediated immunity, and showed that those patients who demonstrated seroconversion to all three vaccine strains had baseline CD38+CD4+ T cell and CD38+CD8+ T cell subpopulations. CD38 is a pleiotropic surface molecule on lymphocytes, and may act as both a receptor and an ectoenzyme. Ligation of CD38 on T cell surface can induce T cell activation and cytokine production [28]. Activation of CD38 can also prevent human germinal center B cells from undergoing apoptosis [29], and thus may potentially explain why patients with increased numbers of CD38+ T cells pre-vaccination demonstrated enhanced seroconversion to all vaccine strains in our study.

Our study, which is one of the first to characterize T cell subpopulations, showed that age-adjusted percentage of CD38+ naïve CD4+ T cells (CD38+CD45RA+CCR7+CD4+ T cells), naïve CD4+ T cells (CD28+CD45RA+CCR7+CD4+ T cells), and senescence central memory CD8+ T cells (CD57+CD45RA-CCR7+CD8+ T cells) were higher in patients treated with HDF compared to those patients treated with HD. T helper cells (CD4+ T cells) play a major role in adaptive immunity, so a loss of CD4+ T cells or functionality might potentially affect both cell-mediated and humoral immunity. CD28 is a co-stimulatory molecule that plays pivotal roles in T cell activation, and is also a marker of the proliferative history of cells. Loss of CD28 expression is a characteristic of the age-associated decline of CD4+ T cell function [30]. Lower CD28+CD45RA+CCR7+CD4+ T cell proportions in the HD group might suggest a relative decline of cellular immune response due to the decrease in the number of naïve T cells. Higher CD57+CD45RA-CCR7+CD8+ T cells in patients treated with HDF might be explained by longer dialysis vintage. Xiaoyan and coworkers, compared ESRD patients treated with continuous ambulatory peritoneal dialysis (CAPD) and hemodialysis, demonstrated that dialysis modality and age influenced T cell subsets [31]. There was a progression from naïve to effector T cells in hemodialysis patients compared with CAPD patients. Another study showed that, comparing to peritoneal dialysis, hemodialysis was associated with higher CD4+CD57+CD28- T cell frequency in CMV-exposed patients [32]. It was hypothesized that sustained repeated antigenic stimulation of T cells during hemodialysis may cause accelerated aging compared to peritoneal dialysis.

The effects of HDF on immunomodulation have not been well characterized. Two previous studies reported a lower production of inflammatory cytokines in dialysis patients treated with HDF [33], however, clinical outcomes of patients were not different [33]. Valkenburg and coworkers reported that influenza-specific CD4+ and CD8+ effector memory T cells produced anti-viral cytokines and these had a protective effect [33]. We did not perform cytokine assay, but we demonstrated higher lymphocyte proliferation to some of the circulating strains of influenza A in the HDF group. This potentially could have been that those patients in the HDF group may have previously been more exposed to influenza infection or immunization. Alternatively, increasing middle-sized azotemic toxins clearance might reduce immune dysregulation associated with CKD, resulting in greater effector and memory T cell function. Previous studies in healthy patients have reported that increased lymphocyte proliferation does not necessarily always correlate with greater antibody responses to vaccinations [34]. We also noted that in our dialysis patients that lymphocyte proliferation did not always associate with seroprotection and seroconversion.

The strengths of our prospective observational study were a detailed analysis, including assessments of both humoral and cellular immune responses to influenza A vaccinations. We accept that our study population was limited, and patients were not randomized to dialysis modality.

## Conclusions

In conclusion, we demonstrated that seroconversion to all three influenza A vaccine strains was influenced by age. Patients with seroconversion to all three influenza vaccine strains were found to have higher CD38+CD4+ T cells pre-vaccination. Seroprotection was better sustained, and lymphocyte proliferation in response to influenza A was greater in dialysis patients treated by HDF compared to those treated by HD. Our study suggests that the immune dysregulation encountered in dialysis patients is reduced in patients treated with HDF, who have a greater clearance of middle-sized azotemic solutes results. These encouraging results require confirmation in larger patient cohorts.

## Supporting information

S1 Fig Boxplots of hemagglutination inhibition (HI) titer in patients treated with HD and HDF pre-vaccination (mo 0), and post-vaccination at months 1 (mo 1), 6 (mo 6), and 12 (mo 12) by influenza strains. (TIFF)

S2 Fig Age-adjusted percentage of memory B cells at pre-vaccination and post-vaccination. (TIFF)

S3 Fig Percentage of virus-stimulated carboxyfluorescein succinimidyl ester (CFSE)-positive lymphocytes at pre-vaccination (month 0), and post-vaccination at months 1, 6, and 12. *P*-values of comparison between pre-vaccination and each time point post-vaccination by Mann-Whitney *U*-test are shown.
(TIFF)
