# Supplementary figures and images for: Immune response to influenza vaccination in ESRD patients undergoing hemodialysis vs. hemodiafiltration
Source: PLoS One. 2020 Feb 3;15(2):e0227719. doi: 10.1371/journal.pone.0227719 (PMC6996846; doi:10.1371/journal.pone.0227719)

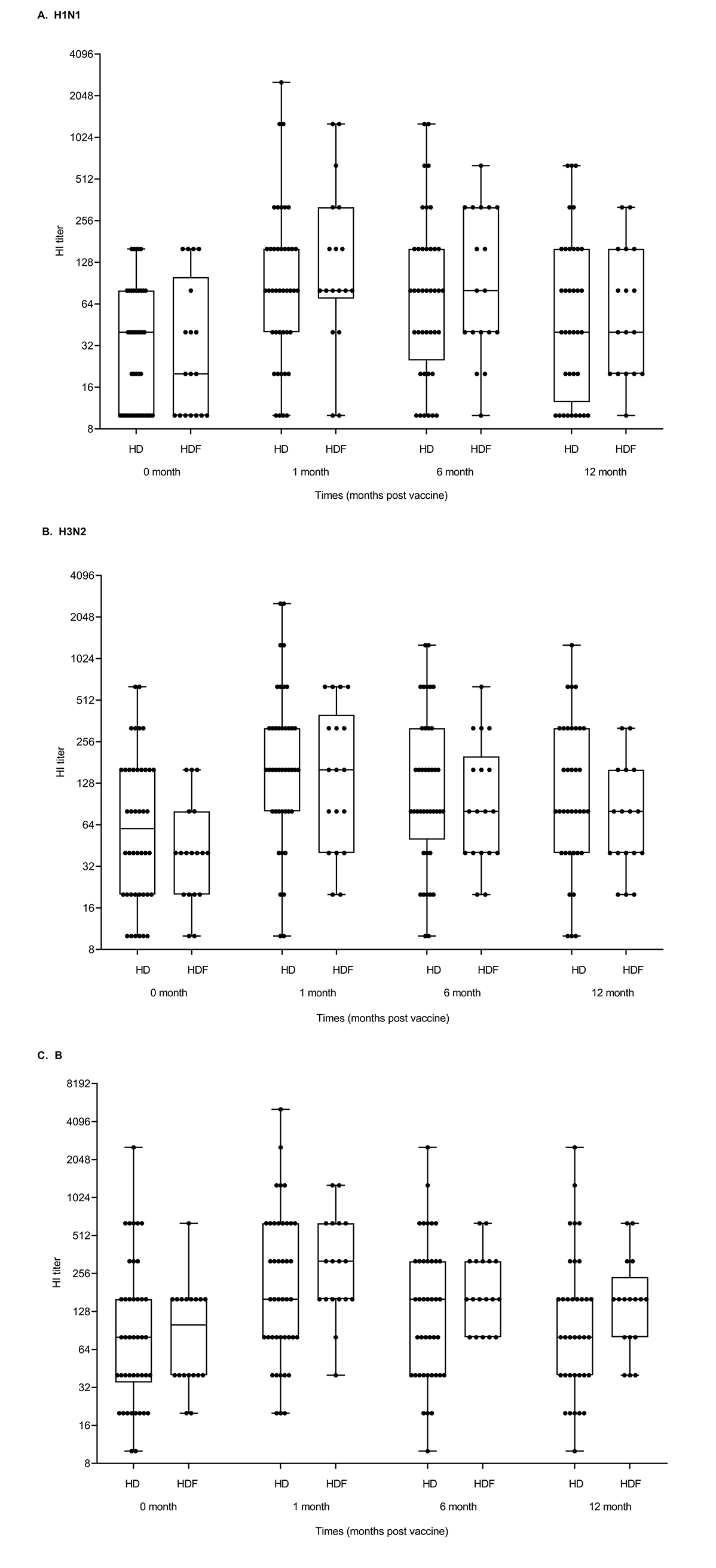

Supplement: S1 Fig — (TIFF) [file pone.0227719.s001.tiff]

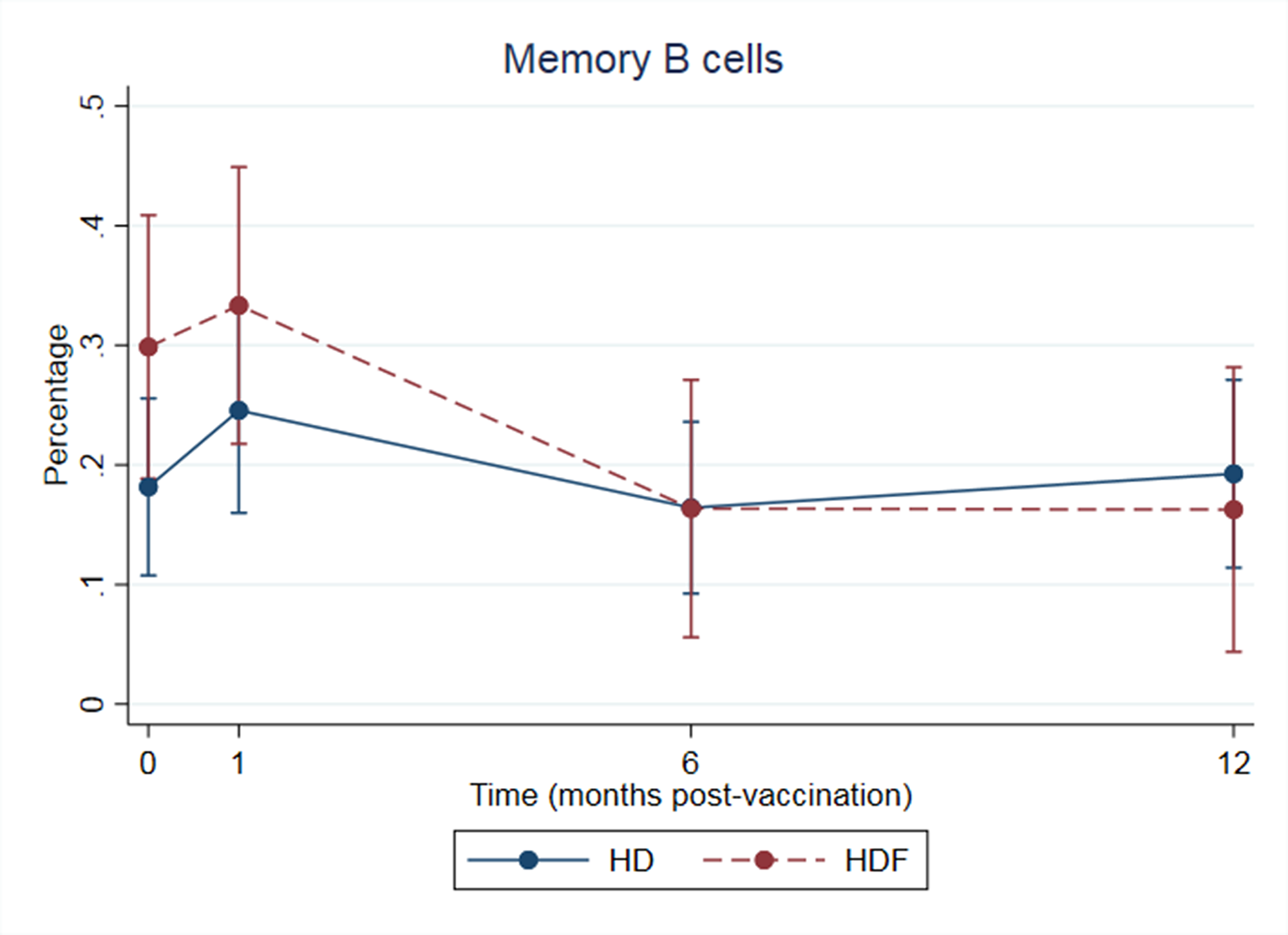

Supplement: S2 Fig — (TIFF) [file pone.0227719.s002.tiff]

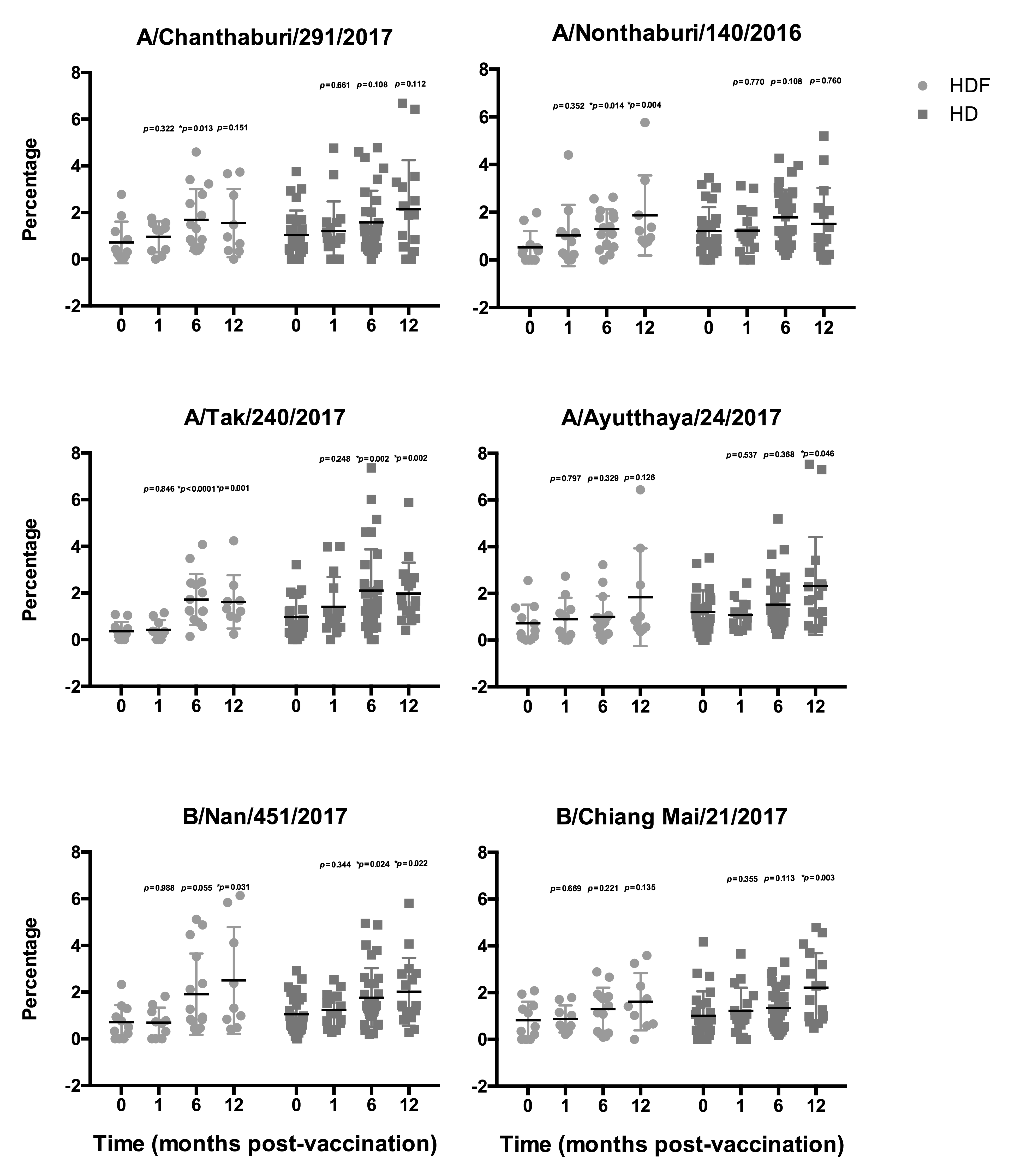

Supplement: S3 Fig — P-values of comparison between pre-vaccination and each time point post-vaccination by Mann-Whitney U-test are shown. (TIFF) [file pone.0227719.s003.tiff]
